# A Rare Case of Laryngeal Osteosarcoma Causing Diagnostic Challenges

**DOI:** 10.7759/cureus.37393

**Published:** 2023-04-10

**Authors:** Yusuf Ç Kumbul, Vural Akın, Hasan Yasan, Gizem Özdoğan Yılmaz, Veysel A Ayyıldız

**Affiliations:** 1 Otorhinolaryngology and Head and Neck Surgery, Suleyman Demirel University, Isparta, TUR; 2 Medical Pathology, Suleyman Demirel University, Isparta, TUR; 3 Radiology, Suleyman Demirel University, Isparta, TUR

**Keywords:** histopathological examination, sarcomatoid carcinomas, diagnostic challenges, total laryngectomy, laryngeal osteosarcomas

## Abstract

Laryngeal osteosarcomas are extremely rare. They cause diagnostic difficulty for the otolaryngologist and pathologist. Differentiation from sarcomatoid carcinoma is challenging but important, as clinical features and treatment strategies are different. Total laryngectomy is generally the preferred surgical approach for laryngeal osteosarcomas. Since lymph node metastasis is not expected, neck dissection is not needed. In this report, we present a case diagnosed with laryngeal osteosarcoma post the examination of the total laryngectomy specimen of a laryngeal tumor that could not be histopathologically differentiated by punch biopsy.

## Introduction

Larynx sarcomas are extremely rare. They constitute 0.3% to 1% of all laryngeal neoplasms. The most common sarcomas in the larynx are chondrosarcoma and fibrosarcoma, while the rarest is osteosarcoma (OS) [1-5]. Osteosarcomas develop in the head and neck region, most commonly in the mandible [6]. Laryngeal OS was first described in 1942 [2]. This malignancy, which poses a diagnostic challenge for the clinician and pathologist, is important in the differential diagnosis of laryngeal masses because it has different clinical features from classic squamous cell carcinoma of the larynx.

## Case presentation

**Figure 1:**
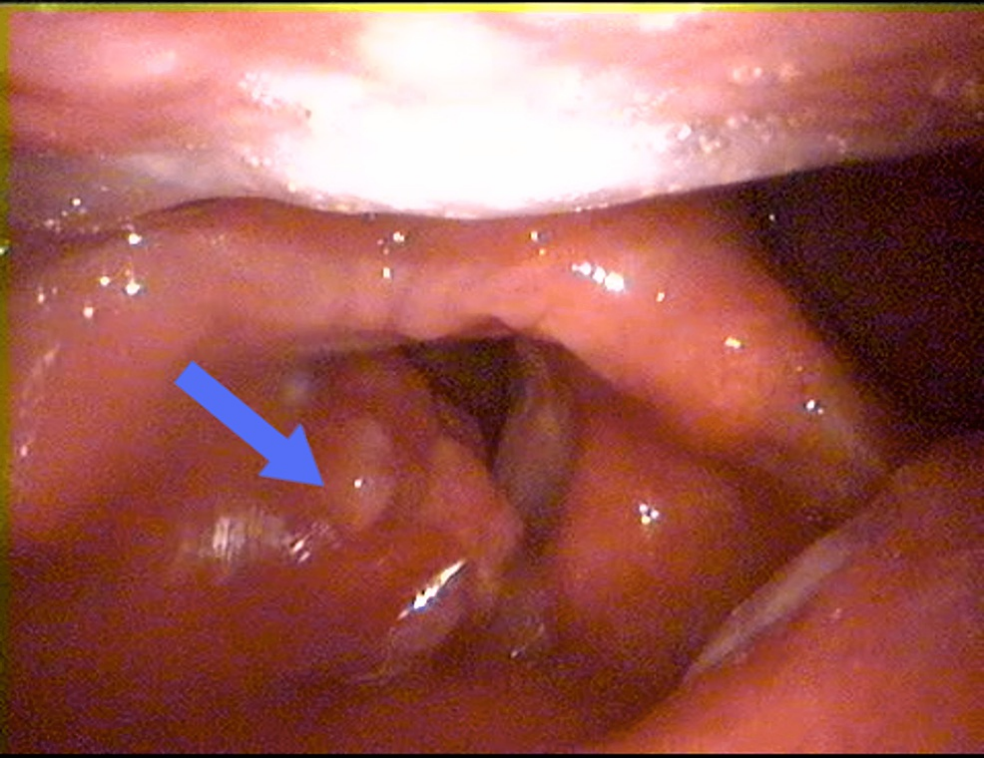
Larynx endoscopy image of the mass A mass is seen involving the right hemilarynx and filling the right supraglottic area (blue arrow).

A 60-year-old male patient was admitted to our clinic with hoarseness and dyspnea for two months. The patient had a history of smoking for 45 years. The patient had no history of alcohol use. In addition, the patient had diagnoses of hyperthyroidism and chronic obstructive pulmonary disease and was not receiving treatment for either disease. There was no history of malignancy in the patient's family history. There was no history of radiation exposure to the head and neck region. Laryngeal endoscopy observed a mass fixed to the right vocal cord that extended from the right vocal cord to the ventricle and anterior commissure (Figure 1).

**Figure 2:**
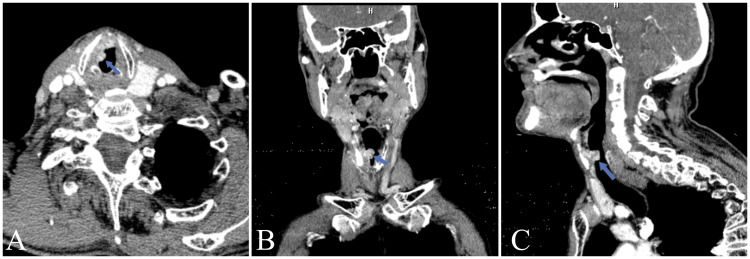
Axial (A), coronal (B), and sagittal (C) images of contrast-enhanced neck CT Originating from the contiguity of the thyroid cartilage at the right glottic level, advancing in the submucosal area, narrowing the laryngeal air column medially, extending to the supraglottic level superiorly, removing the fatty plane between the thyroid cartilage is the mass with a lobulated contour that contains hyperdense areas of calcification and extends anteriorly to the anterior commissure. It is a heterogeneous mass lesion that is 20×11×10 mm in size (AP×TR×CC) with no significant contrast enhancement observed (blue arrows). AP: Anteroposterior, TR: Transverse, CC: Craniocaudal

**Figure 3:**
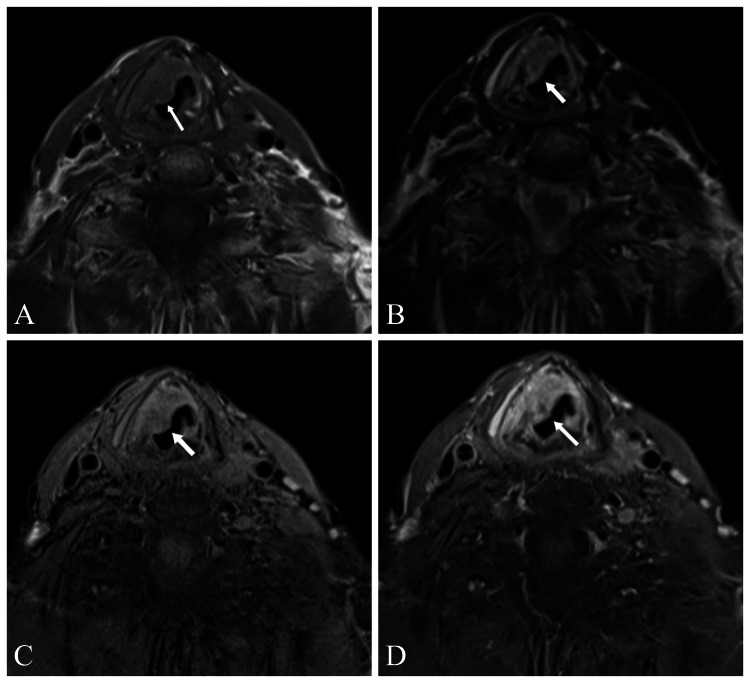
Contrast-enhanced neck MRI of the mass without fat suppression T1-weighted (A), without fat suppression T2-weighted (B), pre-contrast fat suppressed T1-weighted (C), and post-contrast fat-suppressed T1-weighted (D) A homogeneous mass lesion 23×15×12 mm in size (AP×TR×CC) was observed with lobulated contours, hypointense in T1-weighted, hyperintense and heterogeneous in T2-weighted, with homogeneous contrast enhancement in postcontrast T1-weighted fat-suppressed images (white arrows). AP: Anteroposterior, TR: Transverse, CC: Craniocaudal

Contrast-enhanced neck CT and MRI were performed to evaluate the patient in more detail (Figures 2, 3).

**Figure 4:**
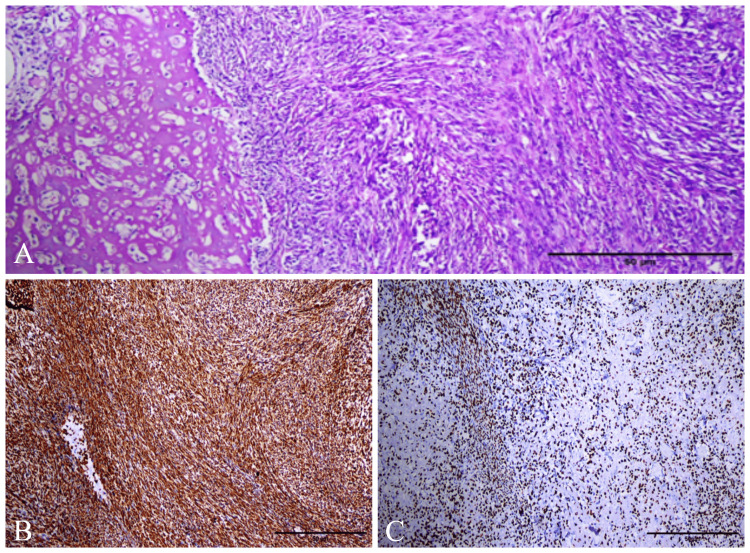
Histopathological examination images of the mass A: Osteoid production and malignant spindle cells in the tumor; B: Diffuse positive expression for vimentin in tumor cells (×100); C: Diffuse positive expression for special AT-rich sequence-binding protein 2 in tumor cells (×100)

It was decided to take a punch biopsy from the laryngeal mass under general anesthesia. With the recommendation of the anesthesia department, treatments for hyperthyroidism and chronic obstructive pulmonary disease were started before general anesthesia. The punch biopsy result was reported as sarcomatoid malignant neoplasia, but sarcomatoid carcinoma and sarcoma could not be differentiated. Locoregional and distant organ metastases were not detected in the patient who was examined with F-18 fluorodeoxyglucose positron emission tomography after the diagnosis of malignancy. As a result of the examinations, it was decided to perform a total laryngectomy and left thyroid lobectomy (with the recommendation of endocrinology). Neck dissection was not planned because the lesion was a possible sarcoma and the neck was evaluated for cN0. The patient was discharged without complications on the eighth postoperative day. Histopathological examination of the total laryngectomy and thyroidectomy specimens were reported as laryngeal OS and multinodular goiter, respectively. The tumor was immunohistochemically positive for vimentin and special AT-rich sequence-binding protein 2, while it was negative for pan-cytokeratin, cytokeratin 19, 7, 20, 18, 5/6, and S100 (Figure 4).

The Ki-67 index for the tumor was 1% to 2% positive. There was no tumor at the surgical margins and no tumor invasion in the thyroid-cricoid cartilages and hyoid bone. There was also no invasion in lymphovascular, perineural, and perilaryngeal tissues. The patient was referred to radiation oncology and medical oncology departments to be evaluated in terms of adjuvant treatments. Monthly otorhinolaryngology department check-ups were recommended to the patient for the first year after surgery. The patient was followed up for three months without locoregional recurrence or distant metastasis.

## Discussion

The median age at diagnosis for head and neck OS is reported to be 38 years with no difference in frequency between the genders [7]. In a study by Mosalleum et al., when laryngeal OSs were evaluated within themselves, the mean age of diagnosis was 63.5 years and was found to develop 13 times more frequently in males than in females [2]. Although laryngeal OS differs from other OSs of the head and neck region in terms of age and gender, it is similar to epithelial carcinomas of the larynx. This is one of the factors that increases diagnostic difficulty. The present case is compatible with the literature in terms of age and gender.

The etiology of a laryngeal OS is still unclear. Radiation exposure history, Paget's disease, fibrous dysplasia, and retinoblastoma are suggested risk factors [2,5]. It was stated that ossification of the laryngeal cartilage might also increase the risk [4]. No relationship was found between cigarette and/or alcohol consumption and laryngeal OS [1,3,8]. However, cigarette and/or alcohol consumption are well-known risk factors for laryngeal carcinosarcoma/sarcomatoid carcinomas, which are important in the differential diagnosis [9]. In the literature review by Mosalleum et al., four of the 25 laryngeal OS cases had a history of radiotherapy and one had Paget's disease [2]. In our case, the above-mentioned risk factors were not present. However, our patient had a history of smoking for many years. Studies with more participants are needed to determine whether smoking is a risk factor for the development of laryngeal OS.

The most common symptoms of laryngeal OS are hoarseness and dyspnea [2]. The most frequently involved areas are the vocal cords, cricoid cartilage, and thyroid cartilage [1,8]. A polypoid-like, cartilage-invading mass that adheres to the thyroid cartilage perichondrium in the anterior commissure without direct extension to the vocal cords is one of the common clinical images in laryngeal OS [2]. In our patient, the endoscopic view during the first attendance was seen as a mass that fills the right hemilarynx. During the period between the first attendance and total laryngectomy, it was noticed that this mass grew polypoidally towards the trachea, filled the tracheal lumen almost completely, had a cheese-like structure, and ruptured easily. Although the hematogenous spread of sarcomas is known, it seems logical that tumor cells separated from the tumor in laryngeal OS can reach the lung by direct transplantation. Perhaps this may contribute to the frequent metastasis of laryngeal OS to the lung [1,5].

The presence of malignant osteoid production and absence of epithelial differentiation suggest OS in the diagnosis. For the diagnosis of OS, epithelial differentiation especially must be excluded [2,9]. It is difficult to distinguish laryngeal OSs clinically, radiologically, and histologically from sarcomatoid carcinomas, metastasis of another sarcoma, and other malignant tumors including osseous metaplasia [2,9]. In addition, chondrosarcoma, malignant fibrous histiocytoma, fibrosarcoma, and laryngeal myositis ossificans should be considered in the differential diagnosis [1]. Microscopically, OS has malignant, spindle-shaped mesenchymal cells associated with osteoid and immature neoplastic bone formation. In the immunohistochemical examination, vimentin is positive, while desmin, S-100, cytokeratin, and epithelial membrane antigen are negative [1]. The case we presented had histopathologically similar staining patterns.

Lymph node metastases in OSs are extremely rare [1,5]. Although there was no clinical lymph node metastasis in our case, no neck metastasis was observed during the three-month follow-up. Due to the extremely rare occurrence of laryngeal OS, it is difficult to develop a specific treatment protocol [1,4,8,9]. Considering that OS may progress submucosal in particular, we believe the chance of achieving a cure with partial laryngectomy is very difficult. In line with our opinion, total laryngectomy is the most frequently preferred surgical approach in the literature [4,9]. Adjuvant chemotherapy may be chosen, but the evidence for the efficacy of radiotherapy is limited [1,2,8]. The most commonly used drugs in chemotherapy are methotrexate, adriamycin, and cisplatin [8]. In Mosalleum et al.'s study, the two-year survival rate was found to be 23.5% and the mean survival was 12.6 months [2].

## Conclusions

Laryngeal OSs are very rare and have an aggressive course. It is particularly important to distinguish them from sarcomatoid carcinomas of the larynx because clinical features and treatment strategies are different. Total laryngectomy is the most commonly recommended treatment for laryngeal OS and the patient should be closely monitored for long-term lung and other organ metastases.
